# Possible new options and benefits to detect myocarditis, right ventricular remodeling and coronary anomalies by echocardiography in systematic preparticipation screening of athletes

**DOI:** 10.1007/s10554-020-01899-1

**Published:** 2020-05-27

**Authors:** Tom Döbel, Stephan Stöbe, Robert Percy Marshall, Pierre Hepp, Sven Fikenzer, Kati Fikenzer, Sandra Tautenhahn, Ulrich Laufs, Andreas Hagendorff

**Affiliations:** 1 University Hospital Leipzig Department for Internal Medicine Neurology and Dermatology, Department of Cardiology, University Hospital Leipzig, Leipzig, Sachsen Germany; 2 RasenBallsport Leipzig GmbH, Leipzig, Germany; 3 Department of Orthopedics, University Hospital Leipzig, Leipzig, Sachsen Germany

**Keywords:** Transthoracic echocardiography, Myocarditis, Sudden cardiac death, Left ventricular remodeling, Cardiac imaging

## Abstract

Exclusion of cardiac abnormalities should be performed at the beginning of the athlete’s career. Myocarditis, right ventricular remodeling and coronary anomalies are well-known causes of life-threatening events of athletes, major cardiovascular events and sudden cardiac death. The feasibility of an extended comprehensive echocardiographic protocol for the detection of structural cardiac abnormalities in athletes should be tested. This standardized protocol of transthoracic echocardiography includes two- and three-dimensional imaging, tissue Doppler imaging, and coronary artery scanning. Post processing was performed for deformation analysis of all compounds including layer strain. During 2017 and 2018, the feasibility of successful image acquisition and post processing analysis was retrospectively analyzed in 54 male elite athletes. In addition, noticeable findings inside the analyzed cohort are described. The extended image acquisition and data analyzing was feasible from 74 to 100%, depending on the used modalities. One case of myocarditis was detected in the present cohort. Coronary anomalies were not found. Right ventricular size and function were within normal ranges. Isovolumetric right ventricular relaxation time showed significant regional differences. One case of hypertrophic cardiomyopathy and two subjects with bicuspid aortic valves were found. Due to the excessive cardiac stress in highly competitive sports, high-quality and precise screening modalities are necessary, especially with respect to acquired cardiac diseases like acute myocarditis and pathological changes of left ventricular and RV geometry. The documented feasibility of the proposed extended protocol underlines the suitability to detect distinct morphological and functional cardiac alterations and documents the potential added value of a comprehensive echocardiography.

## Introduction

An athlete’s motivation to achieve or exceed performance limit is linked to the disposition to maximum effort during excessive sportive activities. In consequence, the cardiovascular system is exposed to exceptional stress in the context of training and competition [1, 2]. However, high-performance sports exertion may be accompanied in athletes in the presence of pre-existing structural or acquired cardiovascular alterations with additional risk with acute life-threatening events and/or negative prognostic impact [3]. Life-threatening cardiovascular events are undoubtedly linked to unknown cardiac structural abnormalities and/or acute acquired cardiac affections. Thus, the primary intention for pre-participation screening (PPS) is the detection of clinically silent cardiac diseases being associated with sudden cardiac death (SCD) or other major cardiac events [4–6].

Systematic strategies should be implemented into the surveillance of competitive athletes to prevent life-threatening cardiovascular events [4, 7]. However, there is no general accepted concept in echocardiography, whether and how to check and to monitor competitive athletes by this imaging technique [8, 9].

The incidence of life-threatening cardiovascular events including SCD in competitive athletes is reported in literature with a range from 0.76 per 100.000 to 13 per 100.000 [10–13]. There are specific recommendations for appropriate screening modalities in athletes by AHA/ACC [14], EHRA/EAPCR [4] and the International Olympic Committee [9]. Generally, the athlete´s medical history as well as physical examination and resting electrocardiogram (ECG) are elements of the PPS. Transthoracic echocardiography (TTE) as a first-line screening tool is only requested by specified sports federations, like the Fédération Internationale de Football Association (FIFA), Union of European Football Associations (UEFA) and the Union Cycliste Internationale (UCI) [4].

Hereditary structural heart diseases and channelopathies represent the most common life-threatening situation in young athletes [15–17]. In contrast, coronary artery disease and acquired heart diseases become more important with age—especially in the field of leisure sports [18, 19]. In general, several cardiovascular pathologies can obviously be detected by imaging modalities like TTE, e.g. dilative, hypertrophic or non-compaction cardiomyopathy, right ventricular (RV) dysplasia, pericarditis and myocarditis, left ventricular (LV) and right ventricular hypertrophy, aortic valve (AV) and aortic root abnormalities as well as coronary anomalies [17]. However, reliable echocardiographic imaging techniques to detect inflammatory cardiac diseases, regional RV dysfunction and coronary anomalies are rarely reported so far. The uniqueness of the present study is to focus predominantly on the detection of these cardiovascular disorders with an increased risk of life-threatening events, which are normally not linked to ECG changes. Increased costs and lack of expertise are the major two reasons for the absence of TTE and/or cardiac magnetic resonance (CMR) in the diagnostic algorithms for athletes [4]. Extended standardized TTE might contribute to improve existing screening modalities [20].

The aim of this study was to introduce an extended TTE protocol for athletes to improve the detection of the mentioned cardiovascular disorders—especially for acquired cardiac diseases—and to test its feasibility in athletes. It was not the intention to test the feasibility of the advanced TEE protocol in a general diverse cohort of all patients in our department of cardiology with obviously more limitations of the acoustic window. By confirming the hypothesis that the extended imaging protocol is feasible, the expected clinical impact of this proposed imaging strategy might contribute to an improvement of the screening tools in sportsmen.

## Materials and methods

### Study population

In the present retrospective single-center study, TTE data sets of 54 male athletes (mean age 24 ± 5) were analyzed. The study group included 29 soccer players and 25 handball players of the major national leagues, who applied to the cardiological outpatient clinic for the pre-season medical check-up every year. All professional sportsmen of the teams were included. No athlete was excluded or refused to consent in the research. Because only male athletes were supervised in our department of cardiology, no female athletes could be included. Informed consent was obtained by the athletes as well as by the responsible staff. All investigations were performed between July 2017 and August 2018 by two physicians having extensive expertise in TTE.

### Echocardiographic evaluation

No significant pathological findings of ECG check, and laboratory blood analyses and physical examinations were documented in all athletes prior to TTE examination in other supervising institutions. TTE examinations in athletes were performed with GE Vivid E9 and E95 systems using M5S/V6 or M5Sc/VT6 probes (GE Healthcare Vingmed Ultrasound, Horten, Norway). The standardized comprehensive TTE protocol corresponds to national and international recommendations [21–24]. The proposed extended imaging was performed to detect specific relevant findings, especially myocarditis, RV remodeling and coronary anomalies. The major difference between conventional and extended TTE is the additional image acquisition of parasternal short axis views at defined LV levels to analyze deformation and of additional oblique sectional planes to visualize the native coronaries, the aortic root and the thoracic aorta, and the documentation of coronary flow by color-coded cineloops and pulsed wave Doppler spectra. The extended TTE protocol has been established prior to all investigations. The sequelae of the standardized image acquisition are illustrated in Table 1. The feasibility to acquire the additional images was checked by their completeness and their standardization to document the LV and RV cavities as well as the myocardial wall by biplane, triplane and three-dimensional (3D) data sets. In addition, the feasibility of the complete assessment of specific cardiac target structures e.g. the entire RV cavity or visualization of coronary ostia and coronary flow was checked [25, 26].

**Table 1:** Sequelae of imaging sequences and analyzing modalities of the extended TTE protocol

Imaging view and modality (e.g. sectional plane, Doppler-spectrum)	Findings of respective target structures, quantitative assessment of target parameters, and potential post-processing	Quantitative target parameters (unit) and/or qualitative analysis of target structures
		Linear measurement of IVS_d/s_ (mm), LVID_d/s_ (mm), LVPW_d/s_ (mm) Calculated parameters: LV EDV/ESV^†^ (ml), LV EF (%), LV FS (%), LVM^‡^ (g), RWT^§^, LVMI^¶^ (g/m^2^), LVRI^††^ (g/ml)
	
	
		Qualitative deformation imaging analysis of LV rotation radial strain/strain rate circumferential strain/strain rate radial strain/strain rate rotation/rotation rate
	
		Analysis of net-effect of multi-level (apical/basal) rotation patterns: twist/twist rate
		Linear measurement of: RVOT_1/2_ (mm) Qualitative analysis of: AV cuspidity
		Qualitative analysis of: Flow conditions of RVOT and proximal pulmonary artery; PV regurgitation
		Quantitative analysis of RVOT V_max_ (m/s), RVOT PG (mmHg), RVOT VTI (cm) Calculation of: RV SV^‡‡^ (ml)
		Visual qualitative wall motion analysis and Deformation imaging analysis of longitudinal LV strain: LV wall motions patterns of anteroseptal and posterior wall GLS^§§^ (%)
		Qualitative analysis of Flow conditions of LVOT and AV
		Quantitative analysis of: LVOT V_max_ (m/s), LVOT PG (mmHg), LVOT VTI (cm), LV SV^¶¶^ (ml), LV CO^†††^ (ml/m^2^)
		Qualitative analysis of Flow conditions of MV
		Quantitative analysis of E wave velocity (m/s), A wave velocity (m/s), MV deceleration time (ms) Calculation of: E/A
		Quantitative analysis of myocardial LV velocities Documentation of LV synchronicity posterior versus anteroseptal
		Qualitatve analysis of anterior RV wall motion Detection of wall motion abnormalities
		Quantitative analysis of anterior myocardial RV velocities Qualitative documentation of RV-IVRT anterior
		Quantitative analysis of IVRT_aLAX_ (ms)
		Linear measurement of LV area_d/s_ (mm^2^), LV length (mm), LA area_d/s_ (mm^2^) Calculation of: LV EDV/ESV^‡‡‡^ (ml), LV EF (%) GLS^§§^ (%)
		Quantitative analysis of myocardial LV velocities Documentation of LV synchronicity inferior versus anterior
		Qualitatve analysis of inferior RV wall motion Detection of wall motion abnormalities
		Quantitative analysis of inferior myocardial RV velocities Qualitative documentation of RV-IVRT inferior
		Quantitative analysis of IVRT_a2C_ (ms)
		Linear measurement of LV area_d/s_ (mm^2^), LV length (mm) Calculation of: LV EDV/ESV^‡‡‡^ (ml), LV EF (%), LA EDV/ESV (ml) Visual qualitative wall motion analysis and Deformation imaging analysis of longitudinal LV strain: LV wall motions patterns of inferoseptal and lateral wall GLS^§§^ (%)
		Quantitative analysis of myocardial LV and RV velocities Documentation of LV synchronicity inferoseptal versus lateral and qualitative documentation of RV-IVRT lateral
		Quantitative analysis of IVRT_a4C_ (ms)
		Measurement of E′ wave velocity septal/lateral (cm/s) A′ wave velocity septal/lateral (cm/s) Calculation of: E/E′
		Qualitative analysis of Flow conditions of TV and TV regurgitation
		Quantitative analysis of: TV regurgitation V_max_ (m/s) TV regurgitation PG (mmHg) Calculation of: sPAP (mmHg)
		Linear measurement of: LVOT (mm), VAJ (mm), SoV (mm), STJ (mm), TTA (mm) at mid-systole
		Morphological analysis of: AV and root
		Morphological analysis of: LV
		Morphological analysis of: RV
		Linear measurement of the diameters of Aortic arch Proximal descending aorta
		Qualitative assessment of flow conditions in the aortic arch and proximal descending aorta
		Linear measurement of VC Diameter_ins/exs_ (mm) Calculation of Collapse index
		Imaging of RCA ostium
		Imaging of Mid-RCA course
		Imaging of LMCA ostium
		Imaging of Ostium and proximal course of RCA
		Qualitative analysis of LAD flow conditions
		Determination of LAD maximum diastolic flow velocity
		Qualitative analysis of RCA flow conditions
		Determination of RCA maximum diastolic flow velocity
		Qualitative analysis of RCX flow conditions
		Determination of RCX maximum diastolic flow velocity

pLAX, parasternal long axis view; 2D, two-dimensional; IVS, inter-ventricular septum thickness; d, diastolic; s, systolic; LVID, left ventricular inner diameter; LVPW, left ventricular posterior wall thickness; EDV, end-diastolic volume; ESV, end-systolic volume; EF, ejection fraction; FS, fractional shortening; LVM, left ventricular mass; RWT, relative wall thickness; LVMI, left ventricular mass indexed to body surface area; LVRI, left ventricular remodeling index; pSAX, parasternal short axis view; RVOT, right ventricular outflow tract; AV, aortic valve; PV, pulmonic valve; pw, pulsed wave; V_max_, maximum flow velocitiy; PG, pressure gradient; VTI, velocity–time-integral; RV, right ventricular; SV, stroke volume; aLAX, apical long axis view; GLS, global longitudinal strain; LVOT, left-ventricular outflow tract; LV, left ventricular; CO, cardiac output; MV, mitral valve; TVI, tissue velocity imaging; IVRT, iso-volumetric relaxation time; a2C, apical two-chamber view; LA, left atrial; a4C, apical four-chamber view; TV, tricuspid valve; sPAP, sytolic pulmonary artery pressure; VAJ, ventricular-arterial junction; SoV, Sinus of Valsalva; STJ, sinotubular junction; TAA, tubular ascending aorta; VC, inferior vena cava; ins, inspiration; exs, exspiration; RCA, right coronary artery; LMCA, left main coronary artery; LAD, left anterior descending artery; RCX, circumflex artery

^**†**^Estimated by Teichholz formula

^**‡**^Estimated by Devereux formula

^**§**^RWT = LVPW_d_/LVID_d_

^**¶**^LVMI = LV mass/BSA

^**††**^LVRI = LV mass/LV EDV

^**‡‡**^RV SV = 0.785 × Diameter_RVOT 2_^2^ × RV VTI

^**§§**^Derived from aLAX, a2C and a4C view

^**¶¶**^LV SV = 0.785 × Diameter_LVOT_^2^ x LV VTI

^**†††**^CO = (LV SV * Heart rate)/1000 (l/min)

^**‡‡‡**^a2C and a4C were used for volume estimation by biplane method of disks summation (modified Simpson’s rule)

The first object of the extended TTE protocol is related to LV morphology and function focusing on the detection of cardiomyopathies and (peri-) myocarditis. Besides the conventional methods, anatomical M-Mode analysis was performed with respect to better standardization by post processing. Biplane, triplane and 3D-data sets might improve the assessment of LV geometry to better transparency of the correct sectional planes in post processing analyses. The conventional analysis of LV function, LV volumes and LV ejection fraction (EF) was extended by the assessment of longitudinal, circumferential, radial and rotational deformation as well as optional 3D volume assessment including layer strain analysis. Especially, detection of abnormalities of circumferential strain and LV rotation might be helpful to introduce further diagnostics like CMR to confirm the diagnosis of myocarditis or chest trauma.

The second object of the extended TTE protocol is related to RV morphology and function focusing on stress-induced RV dilatation and fibrosis, RV remodeling or dysplasia, and the accurate assessment of RV volume changes in follow-up investigations. RV size was evaluated by conventional measurements in two-dimensional (2D) parasternal long and short axis views as well as in the apical four chamber view. The extended RV imaging protocol includes the documentation of the anterior, lateral and inferior right ventricular wall by additional apical RV views by tilting the apical LV long axis view (ALAX) as well as the apical two-chamber view into the RV for speckle tracking RV analysis. To analyze the longitudinal deformation and relaxation time of the lateral, anterior and inferior RV wall color-coded tissue velocity imaging (TVI) cineloops as well as pulsed-wave (pw) tissue Doppler spectra of the basal RV myocardial velocities were additionally acquired. Triplane and 3D images for advanced post processing RV volume analysis is possible by the extended protocol.

The third object of the extended TTE protocol is related to the size and function of the left atrium (LA). Beside biplane conventional analysis of LA size and emptying fraction using the apical two- and four chamber view, extended TTE analysis includes assessment of LA deformation and LA volume measurement after triplane and 3D-image acquisition with respect to better standardization.

The fourth object of the extended TTE protocol including the documentation of additional oblique parasternal and suprasternal views as well as 3D data sets is related to the aortic valve (AV) and aortic root to detect anomalies considering aortic valve cuspidity, aortic root ectasia, ectasia of the tubular ascending aorta and of the aortic arch.

The fifth object of the extended TTE protocol is related to the detection of potential coronary anomalies. Specific documentation of both ostia of the coronaries in mono-, biplane and 3D data sets as well as of the main proximal and mid proportions of the coronaries by multiple additional sectional planes were performed using conventional 2D imaging including additional flow visualization of coronary artery flow by color-coded Doppler imaging as well as pw-Doppler spectra.

Representative images of the standardized extended TTE documentation and the consecutive measurements and analyses are illustrated in Table 1.

### Data analysis

Analyses of all echocardiographic parameters as well as the post processing of the echocardiographic data were performed using the EchoPac software (version 202, GE Healthcare Vingmed Ultrasound AS).

Linear measurements of LV wall and LV dimensions were performed according to recent international recommendations [27]. These values provide LV volume calculation using the Teichholz formula, and for LV mass (LVM) calculation using the Devereux formula. The second approach of LV volume calculation was the biplane method of disks summation according to the modified Simpson´s rule. In addition, the indexed parameters relative wall thickness (RWT = [2 × diastolic LV posterior wall thickness]/diastolic LV inner diameter), LV mass index (LVMI = LV mass/body surface area) and LV remodeling index (LVRI = LV mass/LV end-diastolic volume_2D_) were calculated.

The analysis of global longitudinal strain (GLS) values was performed with 2D-speckle-tracking of the standardized apical LV views using the automated function imaging (AFI) software (EchoPac, GE Healthcare).

**Fig. 1:**
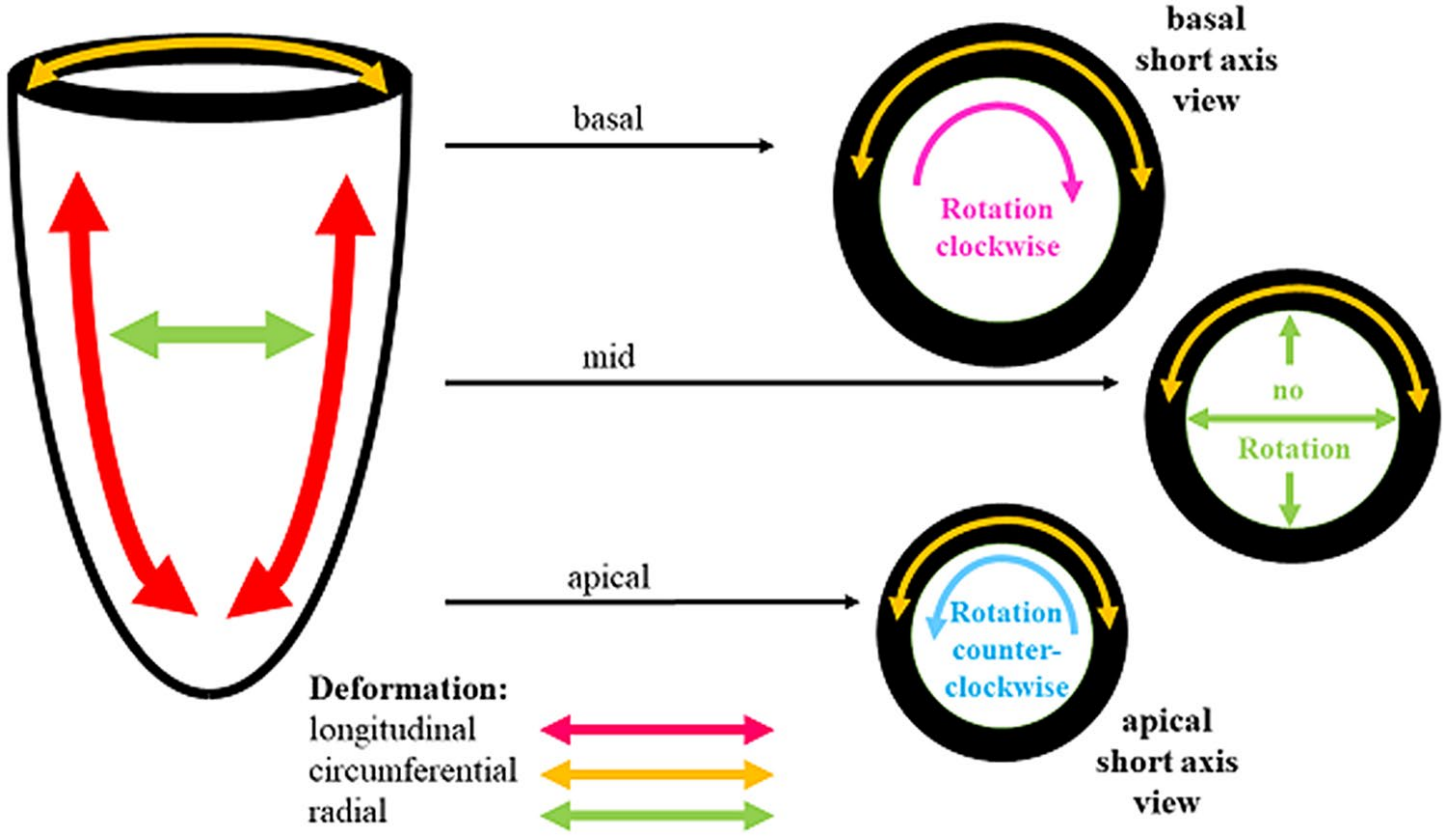
Scheme to illustrate the different deformation parameters: Longitudinal deformation reflects shortening and lengthening of the left ventricular myocardium in direction to the left ventricular long axis. Circumferential deformation reflects shortening and lengthening of the left ventricular myocardium in circular direction illustrated in short axis views of the left ventricle. Radial deformation reflects left ventricular wall thickening in direction perpendicular to longitudinal and circumferential deformation. Twisting and untwisting is characterized by the comparison of the rotation between the base and the apex of the left ventricle, illustrated in short axis views

**Fig. 2:**
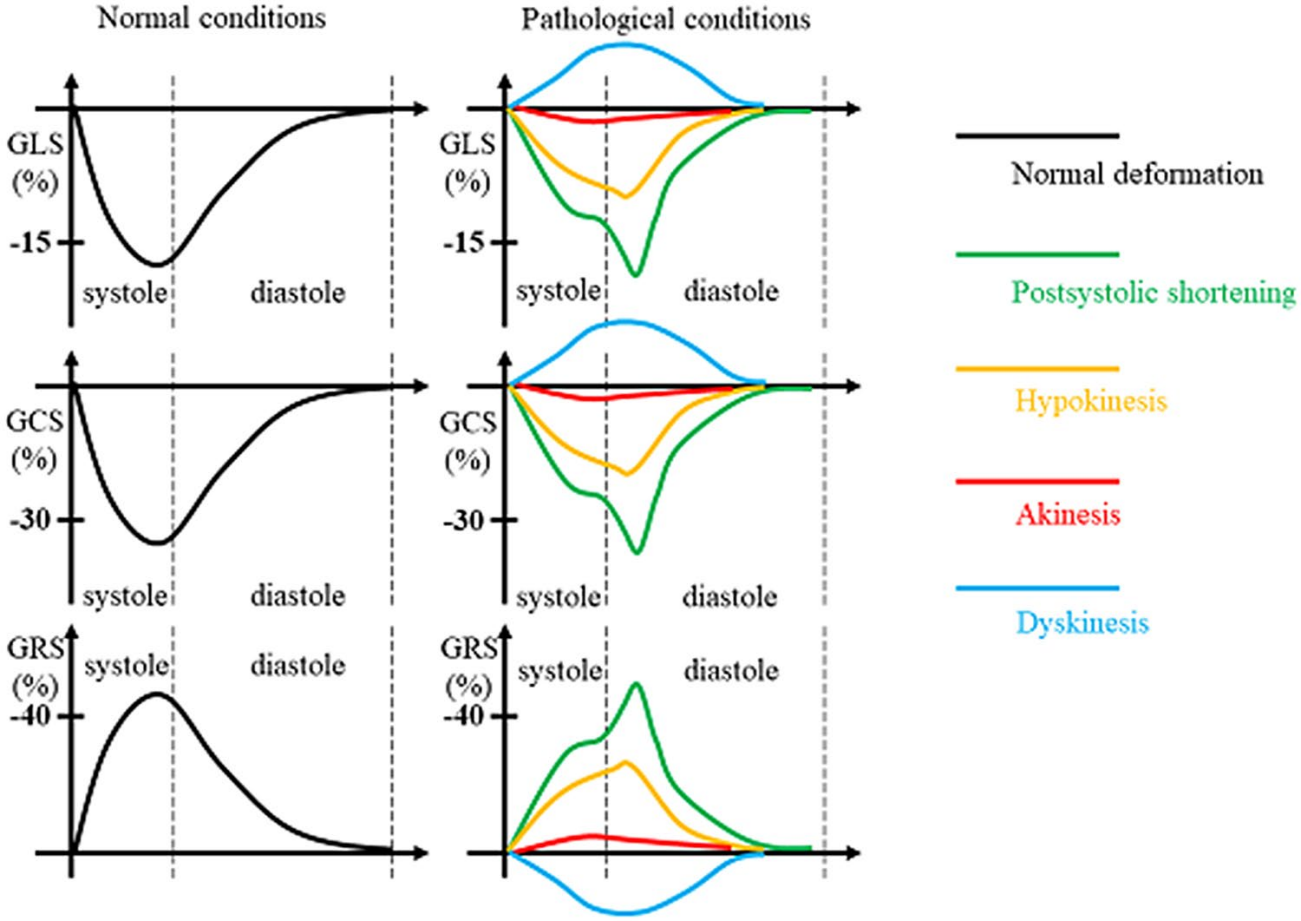
Scheme to illustrate the graphs of normal and pathological deformation of global longitudinal, global circumferential and global radial strain (GLS, GCS, GRS): Normal graphs are shown on the left side, pathological graphs are illustrated at the right side

**Fig. 3:**
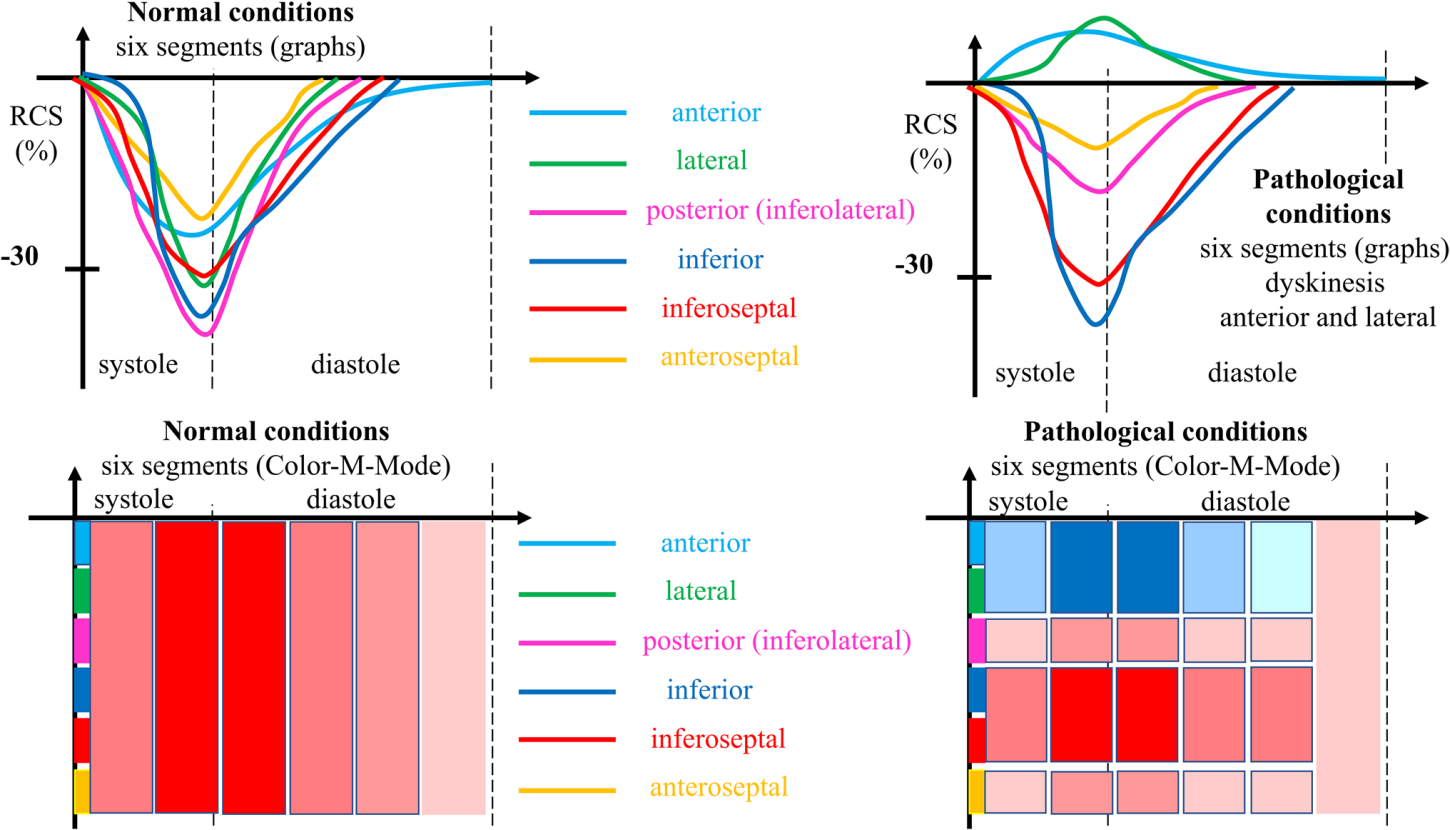
Scheme to illustrate the graphs and color-M-Modes of normal and pathological deformation: Longitudinal or circumferential strain graphs and color-M-Mode schemes were chosen for illustration. The respective graphs of the left ventricular segments are depicted in different colors. The respective left ventricular segments are linked to the color bars at the left side of the color-M-Mode. Normal deformation is illustrated by dark red color at end systole. Pathological deformation is illustrated by the blue color representing dyskinesis

**Fig. 4:**
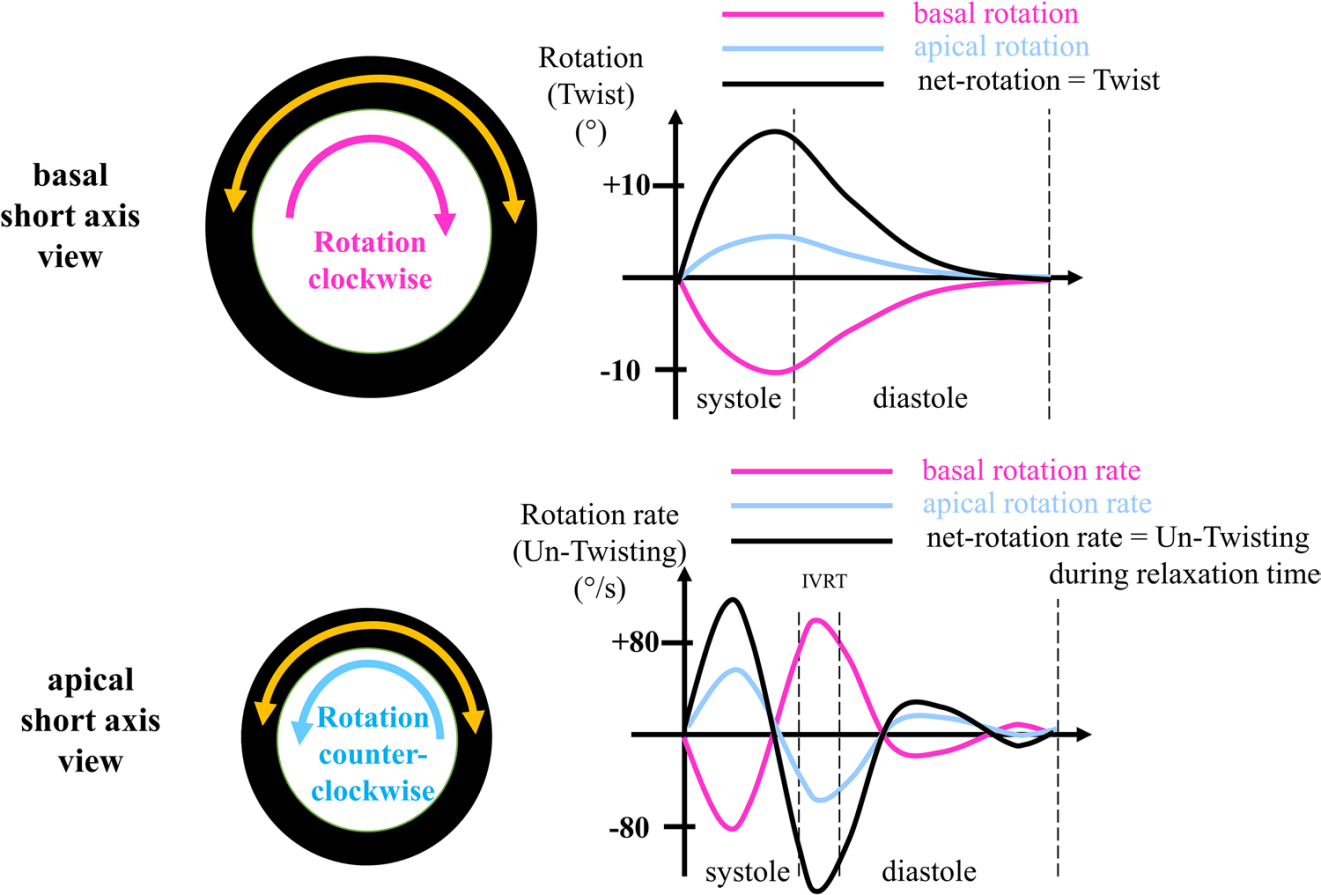
Scheme to illustrate the graphs of rotation and rotation rate: The base is normally rotating clockwise, the apex counterclockwise. Maximum rotation occurs prior to end systole. The graph of normal rotation rate crosses the zero-line prior or at end systole. The amount of systolic twist and diastolic untwisting is illustrated by the net-effect of rotation and rotation rate comparing the basal and apical deformation. *IVRT * isovolumetric relaxation time

The basal, mid and apical parasternal short axis views were used to evaluate circumferential strain and strain rate focusing on layer circumferential strain, radial strain and strain rate as well as rotation and twist using quantitative analysis 2D strain software (EchoPac, GE Healthcare). Especially the comparison of apical and basal rotation expressed as twist and twisting rate were included into the qualitative assessment of rotational deformation abnormalities. The parameters of LV deformation and the distinguishing between normal and pathological conditions are illustrated in Figs. 1, 2, 3, and 4. Figure 1 illustrates the different compounds of LV deformation and the effect on LV rotation during systole at different levels of LV short axis views. Figure 2 illustrates normal and pathological strain curves of longitudinal, circumferential and radial strain. Figure 3 explains the color coding of the strain graphs and of color-M-Modes to illustrate normal versus pathological conditions in a typical example of longitudinal or circumferential strain. Figure 4 illustrates the interpretation of rotational LV function by the graphs of net-rotation and net-rotation rate comparing apical and basal sectional planes.

RV morphology was assessed by conventional measurements of RV dimensions using parasternal short-axis view and modified apical four-chamber view, including linear measurements of the right ventricular outflow tract, RV length, basal-/mid-RV diameter, systolic and diastolic RV area. RV systolic function was conventionally defined with RV fractional area change (RVFAC = [RV area_diastolic_ − RV area_systolic_]/RV area_diastolic_ × 100%). The Doppler analysis of free RV wall was focused on RV relaxation patterns by measuring of regional isovolumic relaxation time (IVRT) at the three appropriate measurement points at the basal free RV wall.

LA volume determination was conventionally performed by biplane disk summation method.

The dimensions of the AV and aortic root were characterized by linear measurements of left ventricular outflow tract (LVOT), ventricular-aortic junction (VAJ) known as aortic anulus, sinus of Valsalva (SoV), sinotubular junction (STJ) and tubular ascending aorta (TAA). The cuspidity was evaluated by 2D- and 3D-data documentation. The AV function was analyzed by color-coded, pw and continuous-wave (cw) Doppler. The LV stroke volume (LVSV) was calculated by M-Mode measurements (LVSV_MMode_), by LV planimetry (LVSV_planimetry_) as well as by Doppler calculation according to the equation: 0.785 × Diameter_LVOT_^2^ × velocity time integral (VTI)_LVOT_ (LVSV_Doppler_). LV cardiac output (CO) was additionally calculated by the equation: LV SV × heart rate.

The coronary arteries were only qualitatively assessed by the successful presentation of the ostia, the proximal and mid proportion of the left main coronary artery (LMCA), the left anterior descending artery (LAD), the circumflex artery (RCX) and the right coronary artery (RCA). In addition, physiological coronary flow was only qualitatively documented.

The triplane and 3D data sets of the cardiac chambers were reviewed for feasible post processing data analysis.

Feasibility of the extended TTE was tested by comparison of conventional cineloops with biplane, triplane and 3D image acquisition regarding standardization of correct sectional planes and complete visualization of the left ventricular myocardium.

### Statistical analysis

All statistical analyses to compare mean values of the alphanumerical data between subgroups were performed using SPSS version 24.0 (SPSS Inc., Chicago, IL, USA). Continuous variables are expressed as mean value ± standard deviation (SD). Group difference verification was performed using the Mann–Whitney U test. A p value < 0.05 was considered to indicate statistical significance. Bonferroni correction was used to adjust the number of comparisons. Thus, p < 0.008 (0.05/59) was considered significant. Power calculation to detect significant changes between means of alphanumeric parameters comparing the subgroups estimates sample sizes for the respective parameters between 13 and 26 individuals, if the significance level is assumed at 0.05 with the test power of 0.9. E.g. estimating a significant GLS difference of 3% at a mean value of about 20% with a SD ± 3% sample size of 26 individuals is calculated.

Categorical parameters, e.g. success rate of imaging, are presented as numbers with their percentages (%). Success rates between the subgroups (soccer vs. handball) were compared using independent sample z-test. The body surface area (BSA), height and height^2.7^ were used for indexing to compare respective values with reference values. A sample size calculation to detect qualitative changes in deformation patterns and the qualitative detection of structural cardiac abnormalities as a criterium of a cardiac pathology can only be performed in comparison to a different imaging method.

The feasibility of the extended TTE protocol as an add-on protocol to the recommended standardized TTE protocol was expressed by the success rates of imaging the respective cardiac structure.

Inter-observer variability of all presented parameters was assessed in ten randomly selected athletes by both experienced investigators using the same datasets blinded to each other’s results.

## Results

### Baseline characteristics

Baseline characteristics of the population analyzed in the present study are summarized in Table 2. A total of 54 male athletes (mean age 24 ± 5 years) were included (29 soccer vs. 25 handball players). All sportsmen had no medication, no history of any cardiac risk factor. No prior sudden cardiac death or other major cardiac events have been explored in the history as well as family history of the athletes. Handball athletes presented significantly larger body dimensions (height, weight, BSA, BMI).

**Table 2:** Baseline charactesristics of the athletes

Parameters (unit)	Total (n = 54)	Soccer (n = 29)	Handball (n = 25)	p value
Age (years)	24 .1 ± 4.7	22.4 ± 3.3	26.2 ± 5.3	0.005
Height (cm)	188 ± 8	184 ± 7	192 ± 7	0.0001*
Weight (kg)	87 ± 12	79 ± 9	96 ± 9	< 0.0001*
BSA (m^2^)	2.1 ± 0.2	2.0 ± 0.1	2.3 ± 0.1	< 0.0001*
BMI (kg/m^2^)	25 ± 2	23 ± 2	26 ± 2	< 0.0001*
HR (1/min)	57 ± 9	57 ± 9	56 ± 9	0.708
Systolic blood pressure (mmHg)	124.0 ± 9.2	125.0 ± 8.5	123.2 ± 9.6	0.90
Diastolic blood pressure (mmHg)	76.7 ± 13.9	66.5 ± 10.0	85.5 ± 10.2	0.19

*BSA* body surface area, *BMI* body mass index, *HR* heart rate, *NS* not significant

*p-value < 0.0008 is indicating statistical significance

### Feasibility of image acquisition, measurements and data analysis

The standard protocol for the 2D TTE echocardiography was completely performed in 53 (98%) subjects. The feasibility of image acquisition and data analysis is listed in Table 3. There was no difference of imaging success rates between the subgroups (soccer vs. handball). Inter-documentation variability of complete image acquisition of the extended TTE protocol did not significantly differ between the two experts. The inter-observer variability of alphanumerical measurements was not statistically different, with a coefficient of variation < 5%.

**Table 3:** Feasibility of image acquisition and data-analysis

Target structure	Analyzing method	Feasibility n (%)
Left ventricular morphology and function	Linear Measurement	54/54 (100%)
	Teichholz volume calculation	54/54 (100%)
	Devereux mass calculation	54/54 (100%)
	Biplane volume calculation	53/54 (98%)
	Longitudinal strain analysis	53/54 (98%)
	Rotational deformation analysis	42/54 (78%)
	Doppler data analysis	54/54 (100%)
RV Morphology and function	Linear Measurement	49/54 (91%)
	Doppler data analysis	43/54 (79%)
Morphology and Function of Aortic valve, root and arch	Linear Measurements	51/54 (94%)
	Doppler data analysis	54/54 (100%)
Coronary anatomy and flow	2D-Visualization of	
	RCA ostium	54/54 (100%)
	Proximal RCA	53/54 (98%)
	Distal RCA	48/54 (89%)
	LMCA Ostium	53/54 (98%)
	Proximal LAD	49/54 (91%)
	Color-Doppler Visualization of	
	Distal LAD	47/54 (87%)
	Distal RCA	46/54 (85%)
	Distal RCX	41/54 (76%)
	pw-Doppler acquisition of	
	Distal LAD	44/54 (81%)
	Distal RCA	43/54 (80%)
	Distal RCX	40/54 (74%)

In addition, the test duration of the advanced TTE approach for the additional image acquisition and the time duration of the post processing analysis take up the time of about 15 min in each athlete. The calculated additional costs of this advanced TTE approach would come up to the amount of 15 min working time of a physician experienced in TTE. However, prerequisites are the logistic availability of the hard- and software to analyze all the additional features, the necessity of transparent standardized image acquisition, and the knowledge about coronary artery scanning.

The biplane LV volume calculation was successful in 53 of 54 (98%) data sets, as well as the LA volume assessment. Linear measurements of cardiac anatomy and LV and LA volume calculation were performed in all athletes. The measurements of the conventional echocardiographic parameters did not show significant differences between both groups, e.g. the left ventricular inner diameter (soccer: 56 ± 3 mm vs. handball: 59 ± 5 mm, NS), LVMI (88 ± 11 g/m^2^ vs. 92 ± 14 g/m^2^, NS) and RWT (0.31 ± 0.04 vs. 0.32 ± 0.07, NS) presented values inside the normal ranges. LV ejection fraction, indexed to BSA, was 62 ± 4% in soccer players and 60 ± 5% in handball players, the LV diastolic function was normal in all subjects, displayed by E wave velocity (0.77 ± 0.11 m/s vs. 0.88 ± 0.42 m/s, NS) and E/E′ ratio (4.9 ± 0.9 vs. 5.2 ± 0.8, NS).

Linear RV dimensions were successfully assessed in 91% of the athletes. There was a significant difference of RV basal diameter (33 ± 4 mm vs. 39 ± 5 mm, p < 0.0001), RV length (80 ± 6 mm vs. 87 ± 5 mm, p < 0.001) and RV systolic area (10 ± 2 cm^2^ vs. 12 ± 2, p < 0.001) between both athlete groups. The functional parameters of the RV showed similar results in both groups with RV FAC (48 ± 4% vs. 47 ± 5%, NS) and tricuspid annular plane systolic excursion (TAPSE, 21 ± 3 mm vs. 23 ± 3 mm, NS). The assessment of relaxation patterns by localized measurements of IVRT was successful 79% up to 96%. No significant difference of IVRT was found between both groups. But regional RV relaxations patterns resulted in significantly shorter IVRT values of the inferior RV wall (37 ± 9 ms) than of the anterior RV wall (45 ± 15 ms), with a p value of 0.003.

Complete assessment of the AV and the aortic root including the proximal parts of tubular ascending aorta was feasible in all athletes. In three athletes the distal ascending aorta and the proximal aortic arch were not adequately visualized for reliable measurements. There was no significant difference of linear aortic measurements between both subgroups of athletes. The values of aortic root diameter (33 ± 3 mm vs. 35 ± 4 mm, NS) and STJ dimension (26 ± 3 mm vs. 27 ± 3 mm, NS) were in the range of the reference values.

Both groups showed CO (4.6 ± 1.2 l/min vs. 27 ± 3 l/min, NS) and CO indexed to BSA (2.3 ± 0.6 l/min/m^2^ vs. 2.2 ± 0.7 l/min/m^2^, NS) values at rest within normal ranges.

The LV deformation imaging analyses of GLS could be performed in all 54 data sets. Analysis of rotational LV deformation could be performed in 78% of the athletes. In two athletes, apical short axis views could not be acquired with adequate image quality. Artifacts, e.g. rib shadowing or lung overlay, were observed in additional 10 data sets of parasternal short axis views.

The coronary artery ostia could be visualized by native 2D echocardiography in all athletes. The feasibility rate for 2D-imaging of proximal proportions of the LAD, RCX and RCA was 98% and 91% respectively. The success rate of coronary flow visualization by color-coded flow imaging (and pw-Doppler) was 87% (81%) for distal LAD, 85% (80%) for distal RCA and 76% (74%) for marginal branches.

### Pathological findings of cardiac morphology and function in PPS

**Fig. 5:**
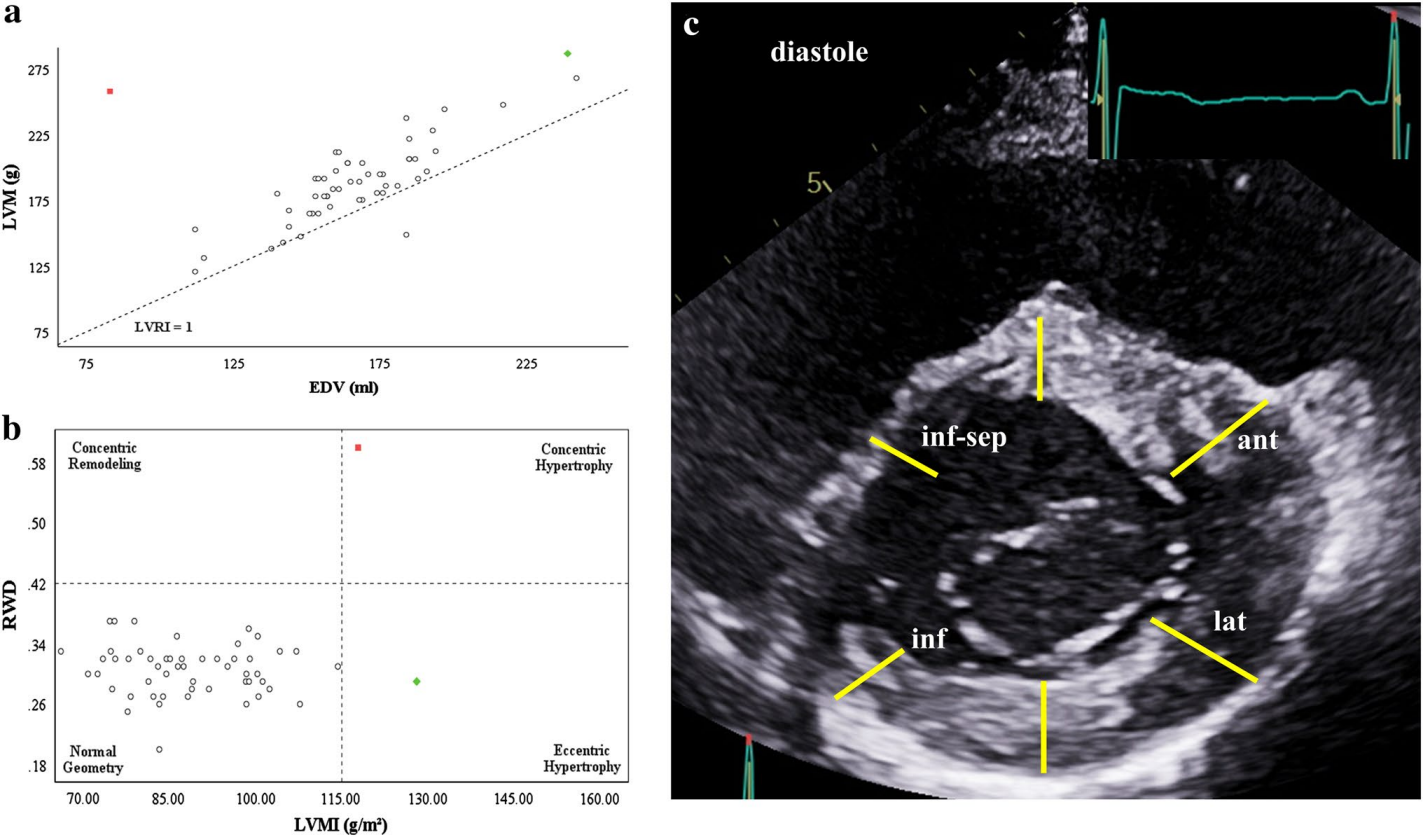
Scattergram of the association between end-diastolic volume (EDV) and LV mass (LVM) (**a**). The illustrated reference line shows the normal relationship between EDV and LVM (LVMI = 1). Scattergram of the association between LV mass index (LVMI) and relative wall thickness (RWT) (**b**): The presented critical values and classification of the LV geometry are derived from Galderisi et al. [18]. Pathological asymmetric LV wall thickening during diastole documents a hypertrophic cardiomyopathy. Yellow bars illustrate the predominant LV thickening in the anterior (ant) and lateral (lat) regions in comparison to the inferior (inf) and inferoseptal (inf-sep) regions (**c**)

**Fig. 6:**
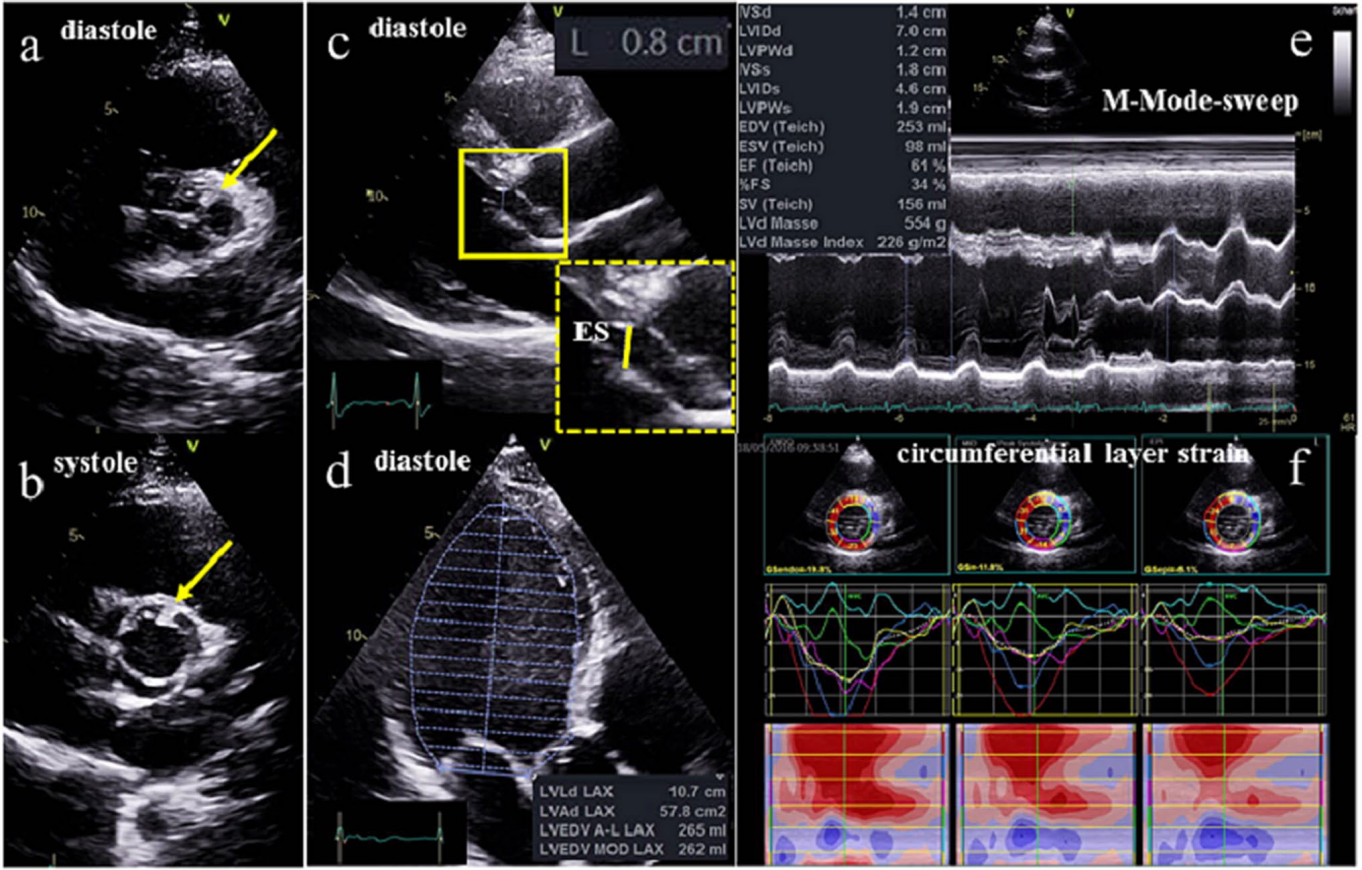
Two-dimensional illustration of bicuspid aortic valve during diastole (**a**) and systole (**b**) in a parasternal short axis view. Illustration of the pathological findings in suspected acute myocarditis confirmed later by CMR. Parasternal long axis view with increase ES-distance during early diastole (**c**), apical long axis view during diastole documenting increased LVEDV of about 265 ml (**d**), M-Mode-Sweep documenting increased end-diastolic LV diameter of 70 mm and increased ES-distance (**e**) and speckle tracking echocardiography of circumferential layer strain (**f**) documenting pathological regional strain of the anterior and lateral LV regions

The relevant echocardiographic findings of LV geometry as well as the documentation of cardiac abnormalities are presented in Figs. 5 and 6.

Figure 5 illustrates the scattergrams between LVM and LV end-diastolic volume (LVEDV) (Fig. 5a) as well as between RWT and LVMI of the athletes to characterize LV geometry (Fig. 5b). There were two subjects with LV geometry outside the normal ranges. Hypertrophic cardiomyopathy (HCM) was described in one subject, identifiable by asymmetric LV hypertrophy (Fig. 5c), who additionally presented a bicuspid AV (BAV). The second athlete fulfilled the criteria of an athlete´s heart (AH) described by an increased LV diameter (68 mm) and LV end-diastolic volume (239 ml), LV mass (LV mass: 287 g, LVMI: 128 g/m^2^) and normal RWT (0.29) and borderline LVRI (1.2), in combination with normal diastolic filling parameters (E/A: 2.1).

In a second athlete BAV without any functional sequels was documented (Fig. 6a, b).

Relevant LV dilatation in the presence of increased LVRI, visually normal LV function and normal GLS was detected in one additional athlete (Fig. 6c–f). However, post-systolic shortening in the apical LV regions and abnormal rotational LV deformation with pathological circumferential strain values as well as pathological twist and untwisting was detected in this case as illustrated in the scheme of Fig. 3. The pathological LV function was also documented by a pathological increase of the early diastolic distance between septum and the anterior mitral leaflets, which would be the only suggesting finding in standard TTE. Due to echocardiographic findings suspected myocarditis was subsequently confirmed by a CMR. One athlete presented a mild aortic regurgitation.

## Discussion

The present study has two important objectives: (1) First, it confirms that an extended TEE protocol is feasible in athletes for PPS, and (2) Secondly, the pathological findings—especially the detection of one case of acute myocarditis—encourage that a more comprehensive TTE approach becomes established in PPS of professional sportsmen.

The feasibility of the described extended TTE approach in athletes was analyzed documenting the following results: successful image acquisition and the post processing analyses of additional sectional planes were above 90%, except for LV deformation imaging (78%), RV IVRT assessment (79%) and the coronary flow detection (74–87%). Relating to the visualization of coronary arteries, several previous studies presented similar feasibility rates [25, 28, 29]. Oxborough et al. [24] recommended the implementation of coronary ostia assessment, but not the analysis of proximal and mid coronary proportions and functional coronary flow Doppler analyses. The existing data about the feasibility of 2D short axis image acquisition for LV deformation analysis are comparable to the present study. However, the influence of artefact on deformation analysis remains unclear [30, 31].

The major question is, if additional pathological findings will be individually detected using an extended TTE protocol compared to the recent standard. While structural cardiac abnormalities like dilative, hypertrophic or non-compaction cardiomyopathy, right ventricular dysplasia, pericarditis, left and right ventricular hypertrophy, AV and aortic root abnormalities can be successfully diagnosed by conventional TTE documentation, the authors believe the proposed extended TTE protocol might be a better alternative for the detection of myocarditis, RV remodeling and coronary anomalies. Thus, higher diagnostic accuracy to detect acquired cardiac diseases—especially in the presence of normal ECG and mild clinical complaints might be provided in follow-up TTE investigations.

LV and RV dimensions, of course, can be correctly determined by conventional techniques using standardized TTE procedures. However, anatomical M-Mode assessment or measurements in biplane/triplane or 3D data sets can document the correctness of the underlying sectional plane. In addition, deformation imaging—especially with respect to circumferential strain—can objectify subtle wall motion abnormalities. Rotational, radial and circumferential parameters might be able to point out pathological findings suspicious for subclinical myocarditis and edema after chest trauma. The significance of layer strain analyses for the detection of acute viral myocarditis should be investigated in further trials [32–35].

A comprehensive conventional as well as the extended TTE must comply with quality criteria of the documentation to ensure the detection of possible distinct pathological findings. The conduction of the TTE investigation by an experienced investigator is one reason for the often-mentioned disadvantages for a first line TTE due to costs and subjectivity of the method [4]. Nevertheless, the PPS programs including TTE in relation to athletes’ outcome is still unknown [36], although morphological and functional cardiac changes in athletes resulting in sport-associated cardiac remodeling cannot be sufficiently monitored by ECG documentation alone because of the lack of specific ECG signs [4, 37].

The excessive stress of the athlete´s cardiovascular system implicates comprehensive diagnostics, especially at the beginning of the competitive career [38]. The presented extended TTE protocol is introduced with the intention to detect important cardiac abnormalities including potential coronary anomalies. The target objectives of the extended TTE can be classified into permanent structural findings and possibly acquired pathologies. Anatomical and structural cardiac findings, e.g. coronary and aortic valve anomalies, will not change under physiological and normal circumstances after the first TTE. However, the extended TTE documentation might be suitable to detect acquired cardiac abnormalities in follow-ups, e.g. due to viral infections. LV and RV function, cardiac valves and the thoracic aorta should be evaluated in every follow-up, because alterations due to infective diseases including (peri-) myocarditis, hypertension and chest trauma can be possible. It is obvious that e.g. the diagnosis of suspected myocarditis cannot be made by TTE findings only. If rotational deformation is documented as abnormal, CMR as the recent gold standard is the next diagnostic step to confirm this diagnosis.

The athlete´s heart adaptation processes in context of different stress mechanisms should be considered [39]. The LV and RV adaptation differs between sports with endurance exercise and strength exercise. Contrary to the incidence of the reported cases of SCD in young competitive athletes, most of SCD occur in non-elite sports. The risk of major cardiovascular events and SCD is heterogeneously reported between competitive versus non-competitive athletes depending on ethnicity, and other associated cardiac risk factors. Thus, the discussion of the relevance of cardiac screening including echocardiography in recreational sports might be reconsidered [8]. In this context, the feasibility of the extended TTE protocol must be confirmed in other patients’ groups, e.g. in older, female and obese individuals.

There is currently a disagreement about the consequences in competitive sport, if relevant pathological findings are detected and whether these findings implicate disqualification criteria for an ongoing sportive career [18, 19]. Considering that the decision of exercise prohibition is normally the end of the athlete’s career, the diagnosis should be clearly confirmed based on diverse diagnostic features and comprehensive expertise is required working with the individual athlete after detecting a relevant finding.

## Study limitations

The major point of the additional value of the proposed TTE protocol in comparison to an ECG-based testing without imaging or a standard comprehensive TTE is hard to analyze in practice. To solve these issue athletes would have to undergo two investigation protocols, e.g. the TTE investigation performed by a non-expert and a second advanced TTE investigation performed by an expert using high-end technology. This is difficult with respect to the time schedule of the athletes in competitive sport. Thus, this comparison should be analyzed in other patients’ cohorts and scenario, which can be done easier in educational projects.

Additionally, investigations would be more meaningful in adolescent athletes at the beginning of their sports career. There were just a few subjects in the present study, which underwent their first comprehensive cardiological screening due to their promotion to professional sports. Moreover, the count of subjects in this study is still too low to derive a significant screening success. The present study concentrates on handball und soccer athletes. Some other sports presenting another profile of requirements should be included in further studies. The heterogeneity of the athletes’ population may limit unifying the message to extend the screening TTE protocol to PPS in all athletes.

## Conclusion

Novel echocardiographic imaging techniques might be useful for the detection of myocarditis, RV remodeling and coronary anomalies. Relevant cardiac pathologies can be successfully diagnosed by the presented TTE screening protocol as documented by the detection of four cardiac abnormalities in 54 subjects. Two findings indicated immediate medical action in terms of further diagnostic and recommendation of temporary/durable abstinence of competitive sports, containing one myocarditis case. Technical feasibility of the presented approach was shown. A possible inclusion of extended TTE screening into the general PPS depends on further investigations in other sports and larger cohorts as well as monetary and educational aspects. Cardiac imaging by TTE as a second line standing in the recent PPS recommendations might be upgraded to a first line tool, relating to the first extensive examination at the beginning of an athlete’s career. Only a well-balanced combination of several diagnostic modalities including TTE might increase the potential to reduce major cardiac events in competitive athletes [9]. The number of detected cardiac abnormalities—especially by post processing analyses—is still too small to establish the present proposal for an extended TTE as a standard, because it cannot be compared whether the observed cardiac pathologies have been detected by a standard conventional protocol. Cardiovascular pathology screening—especially with respect to acute myocarditis and myocardial alterations induced by chest trauma or by excessive exertion—might be more successful using an extended image acquisition as proposed in the present paper [35]. The presented TTE approach sets the stage to determine the prognostic importance of a mandatory repetitive TTE in competitive sportsmen.

## Data Availability

Data can be provided for reasonable request.
